# Sutureless aortic valve replacement in a calcified homograft combined with mitral valve replacement

**DOI:** 10.1186/s13019-017-0642-0

**Published:** 2017-09-07

**Authors:** Ferdi Akca, Kayan Lam, Ibrahim Özdemir, Erwin Tan

**Affiliations:** Department of Cardiothoracic Surgery, Heart Center, Catharina Hospital, Michelangelolaan 2, PO Box 1350, 5602 ZA Eindhoven, The Netherlands

**Keywords:** Sutureless aortic valve, Aortic valve replacement, Homograft, Mitral valve replacement

## Abstract

**Background:**

Aortic valve replacement in a patient with an aortic homograft can be very challenging, especially when concomitant mitral valve surgery needs to be performed.

**Case presentation:**

We report a case of implantation of a sutureless aortic valve bioprosthesis combined with mitral valve replacement in a patient with a severely calcified aortic homograft where conventional valve replacement was technically unfeasible.

**Conclusions:**

We believe that sutureless AVR is a viable option especially for young patients with a high surgical risk where conventional valve replacement cannot be achieved.

## Background

Structural valve deterioration occurs regularly in patients with an aortic root homograft and could require the need for reoperation [1]. This procedure could be technically very challenging with long aortic cross-clamp times, since severe calcifications of the annulus are often present [2]. Furthermore, the operative risk increases with age, multiple comorbidities and the need for concomitant valve surgery. Sutureless aortic valve replacement (AVR) has emerged as a promising technique with good hemodynamic properties and is a viable option for patients with a high surgical risk [3]. Initially, concomitant mitral valve surgery was not recommended with sutureless aortic bioprostheses because of the potential risk of interference at the level of the aorto-mitral continuity. However, more and more reports demonstrate the feasibility of concomitant mitral valve surgery during sutureless AVR [4]. For patients with an aortic root homograft needing reoperation data are scarce. We report a unique case of a patient with a severely calcified homograft who needed concomitant mitral valve replacement (MVR) where conventional AVR was technically unfeasible and implantation of a sutureless aortic bioprosthesis was performed.

## Case presentation

**Fig. 1:**
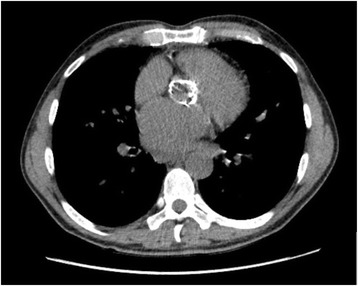
Preoperative computed tomography scan displaying a severe circumferentially calcified aortic annulus

A 55-year old man was hospitalized for congestive heart failure due to combined severe stenosis and regurgitant aortic valve disease. The patient’s medical history included rheumatic heart disease requiring a 23 mm aortic root homograft implantation 21 years earlier. The patient was referred to our center for surgical intervention. Transthoracic echocardiography showed severe aortic valve regurgitation (pressure half time < 200 ms), moderate aortic stenosis (mean gradient 38 mmHg), left ventricular ejection fraction of 0.50 and a moderate mitral regurgitation and stenosis. Coronary angiography showed no significant stenosis, however aortic calcifications were visible and a computed tomography (CT) scan was performed to access the ascending aorta. The CT scan showed severe calcifications at the level of the annulus and distal part of the homograft (Fig. 1). A transcatheter aortic valve implantation was considered, however, due to the young age of the patient, concomitant mitral valve surgery and a strong preference for mechanical valve prostheses the patient was accepted for surgical correction. The calculated logistic EuroSCORE was 5.6% and the Society of Thoracic Surgeons calculated risk of mortality was 3.2%.

**Fig. 2:**
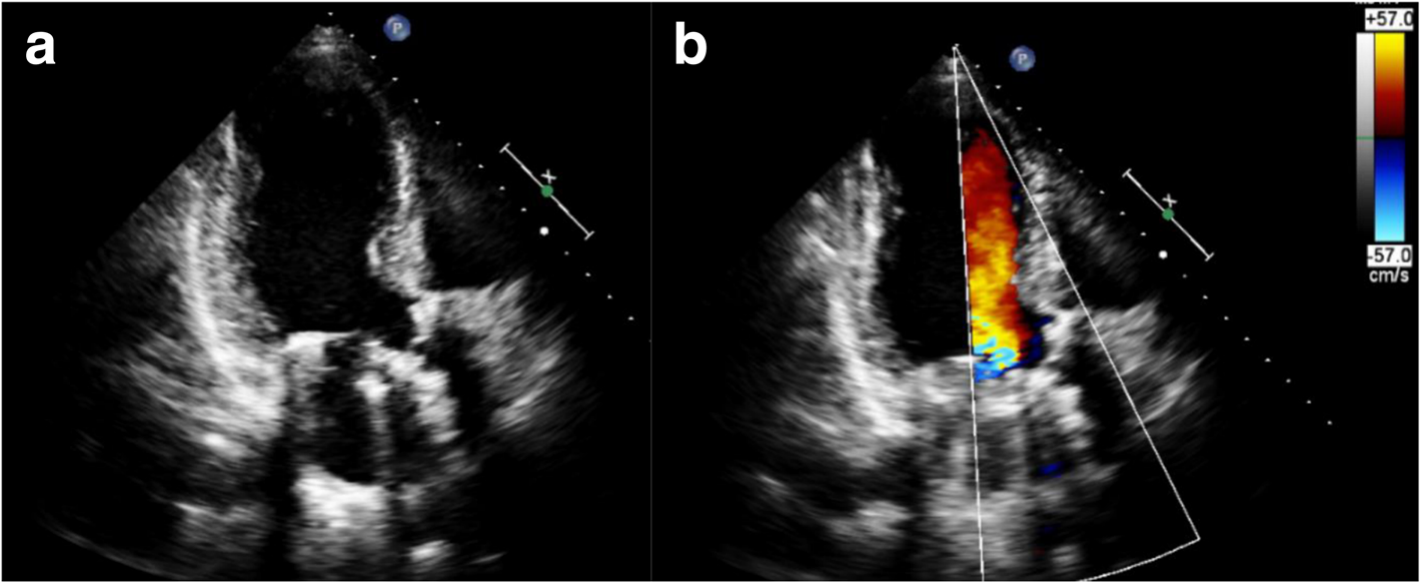
Postoperative transthoracic echocardiography showing the sutureless aortic valve and the mitral valve mechanical prosthesis (**a**) with no sign of perivavular leakage (**b**)

The operation was performed through a median sternotomy and cardiopulmonary bypass was established with cannulation of the proximal aortic arch and bicaval venous cannulation. The body temperature was actively cooled to 33 degrees Celsius. A left ventricular vent was inserted through the right superior pulmonary vein. After clamping the aorta, an oblique aortotomy was made distal of the homograft due to severe aortic calcifications. Custodiol cardioplegia was administered selectively in both coronary ostiae. Sondergaard’s groove was dissected and a rheumatic mitral valve was identified with low likelihood of repair. Therefore, a 31 mm St. Jude Medical Regent mechanical mitral valve prosthesis was implanted (St Jude Medical, Inc., St Paul, Minn, USA). Afterwards, the aortic leaves were excised and extensive decalcification was performed. A conventional mechanical aortic valve replacement was attempted requiring multiple sutures at the annulus. However, due to the extensive degree of calcification these sutures could not be placed. As alternative a colleague skilled with sutureless AVR implantation was consulted. After careful sizing of the aortic annulus a Perceval biological prosthesis of maximal size S was implanted (LivaNova PLC; London, UK) due to circumferential calcifications of the aortic root. Aortic valve placement was performed using three 4–0 Prolene guiding sutures and deployment was successful even with the presence of the mitral valve prosthesis. The aortotomy was closed using a running Prolene 5–0 suture. The cross-clamping time was 190 min and the cardiopulmonary bypass time was 275 min. Intraoperatively transesophageal echocardiogram showed good function of both the aortic and mitral valves with no sign of perivalvular leakage.

The post-operative course was uneventful without the occurrence of any conduction abnormalities. The patient was seen at our outpatient clinic 6 weeks after discharge and recovered well without residual symptoms. A transthoracic echocardiogram was performed after 6 and 12 weeks showing moderate aortic stenosis (mean gradient 29 mmHg) with a indexed effective orifice area of 0.72 cm^2^/m^2^ without any perivalvular leakage (Fig. 2).

## Discussion

Reoperation after homograft implantation could be challenging due to aortic annulus and root calcifications and the procedural risks increase when concomitant valve surgery is performed. Transcatheter aortic valve implantation (TAVI) also developed as a good alternative for patients with a high surgical risk. However, patients with a homograft and predominant aortic regurgitation are a relative contraindication for TAVI. Our patient had combined aortic valve disease, but due to the young age and mitral valve disease surgery was performed. In our patient implantation of a mechanical aortic valve was attempted, however sutures could not be placed in the calcified annulus. Implantation of a sutureless aortic valve bioprosthesis provided a solution even in the presence of a rigid mitral prosthesis. We believe that the use of a St. Jude mechanical mitral valve prosthesis concomitant to sutureless aortic valve implantation is also beneficial as the valve is low profile and will less likely interfere with the Perceval prosthesis. In our patient a high aortotomy was inevitable due to the presence of the calcified homograft facilitating the choice of the Perceval valve, making this the ultimate bail out. Despite the favorable effective orifice area of the sutureless aortic valve a moderate aortic stenosis remained during follow-up resulting in a moderate patient-prosthesis mismatch. This would probably be worse if a stented bioprothesis was used. However, we believe this is the most fortunate outcome of the patient since conventional valve replacement was technically impossible.

## Conclusions

Previous publications reporting on sutureless aortic valve implantation focus mainly on reducing aortic cross-clamp times. Despite the importance of a short cross-clamp time, we implanted the sutureless valve as a last resort option with good success. We believe that homograft redo operation is an ideal indication for sutureless AVR and consider this technique as a viable option especially for patients with a high surgical risk where conventional valve replacement cannot be achieved.

## Abbreviations

AVR: Aortic valve replacement
CT: Computed tomography
MVR: Mitral valve replacement
TAVI: Transcatheter aortic valve implantation
